# Museum DNA reveals the demographic history of the endangered Seychelles warbler

**DOI:** 10.1111/eva.12191

**Published:** 2014-08-11

**Authors:** Lewis G Spurgin, David J Wright, Marco van der Velde, Nigel J Collar, Jan Komdeur, Terry Burke, David S Richardson

**Affiliations:** 1 School of Biological Sciences, University of East AngliaNorwich, Norfolk, UK; 2 Behavioural Ecology and Self-organization Group, Centre for Ecological and Evolutionary Studies, University of GroningenGroningen, The Netherlands; 3 Department of Animal and Plant Sciences, NERC Biomolecular Analysis Facility, University of SheffieldSheffield, UK; 4 BirdLife InternationalCambridge, UK; 5 Nature SeychellesRoche Caiman, Mahé, Republic of Seychelles

**Keywords:** *Acrocephalus sechellensis*, approximate Bayesian computation, bird, bottleneck, island, microsatellite

## Abstract

The importance of evolutionary conservation – how understanding evolutionary forces can help guide conservation decisions – is widely recognized. However, the historical demography of many endangered species is unknown, despite the fact that this can have important implications for contemporary ecological processes and for extinction risk. Here, we reconstruct the population history of the Seychelles warbler (*Acrocephalus sechellensis*) – an ecological model species. By the 1960s, this species was on the brink of extinction, but its previous history is unknown. We used DNA samples from contemporary and museum specimens spanning 140 years to reconstruct bottleneck history. We found a 25% reduction in genetic diversity between museum and contemporary populations, and strong genetic structure. Simulations indicate that the Seychelles warbler was bottlenecked from a large population, with an ancestral *N*_e_ of several thousands falling to <50 within the last century. Such a rapid decline, due to anthropogenic factors, has important implications for extinction risk in the Seychelles warbler, and our results will inform conservation practices. Reconstructing the population history of this species also allows us to better understand patterns of genetic diversity, inbreeding and promiscuity in the contemporary populations. Our approaches can be applied across species to test ecological hypotheses and inform conservation.

## Introduction

Evolutionary processes are often overlooked by biologists and policy makers interested in conserving endangered species. This is a problem, as understanding the demographic history of populations, and thus the evolutionary pressures that they may have faced, is of conservation importance. High levels of inbreeding in small populations can increase homozygosity, and thus the expression of deleterious recessive alleles, with negative fitness consequences (inbreeding depression; Charlesworth and Charlesworth 1987). A second problem in small populations is the loss of allelic diversity at functional genes, which can compromise the ability of a population to adapt to new or changing environments (loss of evolutionary potential; Soulé 1985). Inbreeding depression and loss of evolutionary potential will almost certainly increase the risk of extinction of populations, and by proxy, species (Frankham 1995; Saccheri et al. 1998). One important factor to consider is how long populations have been small: in populations that have experienced continuous, long-term exposure to inbreeding (e.g. small island populations), genetic load can be purged (Crnokrak and Barrett 2002). Although the efficiency with which purging in natural populations reduces genetic load remains uncertain, populations that have been small for a long time may be of less conservation concern than populations that have undergone recent, drastic reductions in population size (Crnokrak and Barrett 2002).

Piecing together the history of wild populations is also of broader biological interest as it helps researchers make sense of present-day behavioural and ecological processes. Historical population bottlenecks, in particular, can affect patterns of individual survival, reproduction and mating behaviour for many subsequent generations, even if the population recovers (Keller et al. 1994; Bijlsma et al. 2000). Some populations may therefore not exhibit the evolutionary responses predicted based on their contemporary demography and selection pressures, due to time-lag effects of historical population declines, expansions or isolation events (Blumstein 2002). The effects of demographic history on these parameters will depend on the timing, extent and duration of the bottleneck (Miller and Hedrick 1991; Briskie and Mackintosh 2004). However, in most cases detailed data on population history are not available, especially for wild species in which historical bottlenecks have rarely been observed or accurately documented.

Genetic markers can be used to provide insights into individual and population-level processes that are not directly observable. At neutral loci, changes in population size leave signatures on patterns of population-level genetic variation, and a number of methods of detecting bottlenecks from genetic data have been developed (Cornuet and Luikart 1996; Garza and Williamson 2001). Recent developments in analytical methods, particularly simulation-based approaches, have enabled researchers to use genetic data to make increasingly complex and detailed inferences about demographic history (Hoban et al. 2012). An especially promising approach is approximate Bayesian computation (ABC) which has been used to infer the timing, duration and severity of bottlenecks and to reconstruct prebottleneck ancestral population sizes from present-day data (Hoffman et al. 2011; Fontaine and Snirc 2012). However, one problem with using present-day DNA samples to study population history is that inferences are still indirect, with the possibility of introducing errors when inferring process from pattern. Different demographic histories can leave similar genetic signatures in contemporary populations and can also mask one another, meaning that key demographic events can be misinterpreted or not detected (Lavery et al. 1996; Schoville et al. 2012). The study of DNA from museum specimens, archaeological finds and fossil remains can address these issues (Hofreiter et al. 2001; Pääbo et al. 2004). By comparing historical and contemporary DNA sequences or markers, studies have been able to assess directly how genetic diversity changes through time in nonmodel organisms. Most of these studies have been restricted to one or a few loci, such as short regions of mitochondrial DNA (Shapiro et al. 2004; Calvignac et al. 2008), microsatellites (Bouzat, Paige, and Lewin 1998; Tucker et al. 2012), or adaptive loci such as the MHC (Smulders et al. 2003). Museum DNA may be especially powerful when used in conjunction with a coalescence-based simulation approach such as ABC (Chan et al. 2006). From a conservation perspective, much of the strength of this approach lies in its use in linking historical and contemporary population changes to anthropogenic impacts, or otherwise, and subsequently directing conservation efforts.

**Figure 1:**
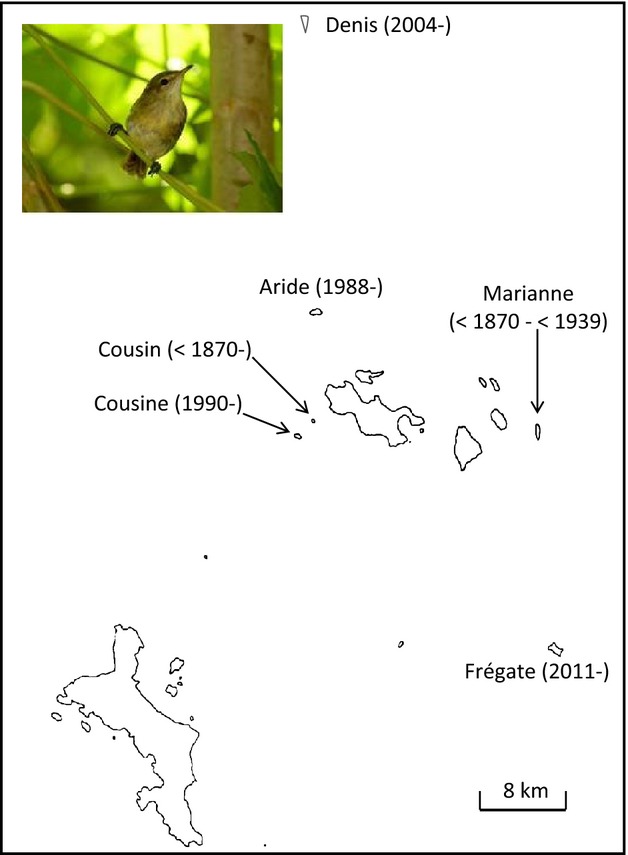
Population history of the Seychelles warbler (pictured inset). Dates represent first dates that Seychelles warblers were present on individual islands, and the last known date on Marianne, where the warbler was known to exist but is now extinct. Note that populations on Cousine, Aride, Denis and Frégate were established by translocations.

Here, we examine how genetic diversity has changed over a period of 140 years in the Seychelles warbler (*Acrocephalus sechellensis*), a small passerine endemic to the Seychelles archipelago in the Indian Ocean (Fig.1). In the 1960s, this species was reduced to a single population of reportedly fewer than 30 individuals on the tiny island of Cousin (4°20′S, 55°40′E, 0.29 km²; Penny 1967; Loustau-Lalanne 1968), and its recovery has been the focus of great conservation attention and scientific interest (Komdeur 1994; Komdeur and Pels 2005; Wright et al. 2014). In the process, the Seychelles warbler has become a long-term ecological and evolutionary model system (Komdeur 1992; Richardson et al. 2003; Barrett et al. 2013), making it an excellent candidate for studying population history. The species was confined to a restricted range (two predator-free islands) in the late 19th century (see Materials and methods), and it is unknown whether it was ever more widespread across the Seychelles, although it is assumed to have been so before the introduction of predators such as rats (*Rattus* spp.) and cats (*Felis catus*) (Collar and Stuart 1985). Relatively low levels of genetic diversity have been observed in the warbler at neutral and functional loci, and this has been linked to fitness (Richardson and Westerdahl 2003; Richardson et al. 2005; Brouwer et al. 2007, 2010). Despite this, the roles of natural and anthropogenic factors in shaping the patterns of genetic diversity in this species remain unknown. We first use DNA extracted from museum specimens to compare microsatellite diversity in the historical population with the contemporary population, allowing us to calculate how much genetic diversity has been lost over time. We then use a Bayesian approach, informed by available knowledge of the warbler's demographic history, to estimate ancestral population size and determine the timing and severity of the bottleneck. We then discuss how natural and anthropogenic factors have shaped patterns of genetic diversity in the Seychelles warbler, as well as the broader implications of using our methods and results for evolutionary and conservation biology.

## Materials and methods

### Study species and sampling

The demographic history of the Seychelles warbler is outlined in Fig.1. The species was first described in 1878 by Oustalet (1878) from the island of Marianne (96 ha), and in the same account was said by Lantz to be ‘rare on Ile Cousine’. Subsequent studies found the warbler on Cousin, but not Cousine, and Lantz's account was presumed to be a mistake (Vesey-Fitzgerald 1940). By 1938, the warbler was extinct on Marianne, and Vesey-Fitzgerald (1940) remarked that it ‘must be the rarest [bird] in the world’. Expeditions to Cousin in 1959, 1965, 1967 and 1968 documented 30, 50, 26 and 50 individuals, respectively (Penny 1967; Loustau-Lalanne 1968). However, birds were not uniquely ringed during these trips, so these estimates of population size are unlikely to have been very precise. In 1967, Cousin was designated as a nature reserve, and efforts began to increase the populations of native bird species (Penny 1967). Habitat restoration, consisting of the removal of coconut palms (*Cocos nucifera*) to allow the succession of natural pisona (*Pisonia grandis*) woodland, was successful, and the Cousin warbler population quickly recovered; since the 1980s, it has been at a carrying capacity of approximately 320 adults (Brouwer et al. 2009). Between 1987 and 2011, four new warbler populations were successfully established by translocation to the islands of Aride, Cousine, Denis and Frégate (Komdeur 1994; Richardson et al. 2006; Wright et al. 2014).

Historical samples were obtained from all known Seychelles warbler museum specimens, collected from Cousin (*n* = 19) and Marianne (*n* = 7) in 1876–1940 (Table S1). Although the temporal range of sampling of the museum specimens was wide, structure analyses suggested that they grouped into two populations (see Results), enabling us to group them for population genetic analyses. A small (approximately 1.5 × 1.5 × 3.0 mm) piece of skin was excised from the ventral surface of the foot and stored at room temperature in a sterile microfuge tube. Contemporary samples were collected as part of an intensive, long-term study of Seychelles warblers on Cousin Island (Brouwer et al. 2010). Since 1988, the entire population has been extensively monitored, often in both the main (June–September) and minor (November–March) breeding seasons each year, during which birds are routinely caught with mist nets and audio lures. A blood sample (approximately 25 μL) was collected from each bird by brachial venipuncture and stored at room temperature in a screw-topped microfuge tube containing 1.5 mL absolute ethanol. Each bird was fitted with a unique combination of three colour rings and a metal British Trust for Ornithology (BTO) ring. Over 96% of adult birds on Cousin have been ringed since 1997 (Richardson et al. 2001), and a representative sampling of the population was achieved in each year. For the present analysis, 50 samples were randomly chosen from 1997 and 2011 (of 160 and 197 samples available from that year, respectively) to provide two temporally distinct contemporary population samples for comparison with the historical data.

### Molecular methods

Contemporary DNA was extracted using a salt extraction protocol (Richardson et al. 2001). DNA was extracted from the museum samples using a Qiagen DNeasy tissue kit (Qiagen, Crawley, UK) according to the manufacturer's instructions, with the following alterations: (i) each sample was finely chopped in a small volume of ATL buffer prior to digestion with proteinase K; (ii) 20 μL 1 m DTT (Dithiothreitol, Sigma-Aldrich, UK) was added at incubation; and (iii) 1 μL carrier RNA (Qiagen, final concentration = 20 μg/mL) was added during the precipitation phase (Freed and Cann 2006). To reduce the risk of contamination, extractions were performed in separate batches of four, with the incorporation of a negative control at the extraction and PCR stages. All DNA extractions were performed in a laminar flow cabinet in a ‘clean room’ located separate from the main laboratory. No passerine DNA had previously been processed in this facility. All equipment was isolated exclusively for museum sample extraction, regularly cleaned with industrial methylated spirits and UV sterilized; all materials were autoclaved where appropriate.

The potential for amplification of each museum DNA sample was tested by molecular sexing using the Z-002D marker set (Dawson 2008). Each PCR included 2 μL Qiagen PCR multiplex master mix, 1 μL primer mix (at a final concentration of 0.2 μm) and 1 μL DNA. The PCR cycling conditions were 15 min at 95°C, followed by 45 cycles of 30 s at 90°C, 1 min 30 s at 56°C and 1 min 30 s at 72°C. All pre-PCR work was carried out in the ‘clean room’. Each contemporary sample was molecularly sexed and genotyped at 30 polymorphic microsatellite loci combined into four multiplexes (Tables S2 and S3). PCR amplification of the contemporary samples was performed in 10 μL volumes containing 20–50 ng of template DNA, using a Qiagen Multiplex PCR Kit and the manufacturer's protocol. The PCR program used was as follows: 95°C for 15 min, followed by 30 or 35 (for the museum specimens) cycles of 94°C for 30 s, annealing temperature (*T*_a_; multiplex-specific, Table S2) for 90 s and 72°C for 60 s, followed by 60°C for 30 min. All PCR products were separated on a 48-well capillary ABI 3730 DNA analyser, and allele sizes assigned using GeneMapper 4.0 software (Applied Biosystems, Paisley, UK). All samples were genotyped at least twice to assess repeatability, and new alleles (i.e. those that have not been found in the routine genotyping of contemporary individuals during the long-term project) were only confirmed when observed in both reactions. Twelve of the 30 markers were successfully genotyped using the partially fragmented DNA extracted from the museum samples (Table S4), so only the corresponding loci from the contemporary samples were included in the data set used for comparison.

### Statistical analyses

Unless stated otherwise, all plots and statistics were generated in R version 2.14.1 (http://www.r-project.org/). For each locus and population (Marianne museum; Cousin museum, 1997 and 2011), we calculated observed and expected heterozygosity and tested for deviations from Hardy–Weinberg equilibrium (HWE) using GENEPOP version 4.0.10 (Raymond and Rousset 1995). Null allele estimates were calculated in CERVUS version 3.0.3 (Marshall et al. 1998). Allelic richness and number of private alleles in each population were calculated after controlling for differences in sample size, using a rarefaction approach implemented in HPRare (Kalinowski 2005).

To examine patterns of genetic structure across populations, we calculated pairwise *F*_ST_, using Slatkin's transformation (Slatkin 1995). For comparison, we also calculated Jost's *D*_EST_ (Jost 2008) in SMOGD (Crawford 2010). Additionally, we looked for evidence of genetic structure using a Bayesian clustering algorithm implemented in the program STRUCTURE (Pritchard et al. 2000). We used a model allowing admixture and correlated gene frequencies and included sampling locations as prior information to deal with the low sample size on Marianne and with potentially subtle structure within our samples (Kalinowski 2011; Porras-Hurtado et al. 2013). We carried out four independent runs for each value of *K = *1–5. For each run, we used 500 000 steps, with a burn-in of 10 000 steps. The value with the highest mean ‘log probability of data’ was considered the most likely number of clusters. We also inferred the most likely numbers of clusters using the method of Evanno et al. (2005). Finally, we carried out a principal components analysis of the microsatellite data using the ADEGENET package in R (Jombart 2008).

We used two approaches based on summary statistics to detect whether the warbler populations had undergone genetic bottlenecks. We first tested for heterozygosity excess, which occurs owing to the loss of rare alleles shortly after bottlenecks (Cornuet and Luikart 1996), using the program BOTTLENECK (Piry et al. 1999). We used a two-phase mutation model and ran the analysis three times, with the percentage of stepwise mutations set at 95%, 90% and 80%, respectively. The probability of heterozygosity excess was calculated using Wilcoxon rank-sum tests. We also calculated Garza and Williamson's (2001) index (*M*), by dividing the number of alleles in a population (*k*) by the range in allele size (*r*).

We used the program DIY-ABC v2.0 (Cornuet et al. 2008, 2014) to estimate the timing and severity of the Seychelles warbler bottleneck. Because of the low sample size on Marianne, we excluded this population and focused on the temporal samples from Cousin. We constructed a bottleneck scenario, using the following priors: the prebottleneck *N*_e_, postbottleneck *N*_e_ and the timing of the bottleneck (Table1). For comparison, we tested the bottleneck scenario against a null model, which simulated a constant *N*_e_ on Cousin over time (Table1). We assumed a generation time of 4 years, which corresponds to the median age of successful breeders (M. Hammers, unpublished data) and dated the museum population at 26 generations before the 2011 samples (the mid-point of the range of museum sample dates). All priors were given uniform distributions, informed where possible by knowledge of the Seychelles warbler population history. The microsatellite loci were assumed to follow a stepwise mutation model, with mean mutation rate drawn from a uniform prior with minimum and maximum values set at 10^−3^ and 10^−5^, respectively, with individual locus mutation rate drawn from a gamma distribution (mean = mean mutation rate and shape = 2). For each scenario, we simulated 1 million data sets. As summary statistics, we used the mean number of alleles per locus, mean gene diversity and mean Garza–Williamson *M* index within each population, as well as pairwise *F*_ST_ across each pair of populations. The posterior probability of scenarios was estimated by (i) taking the 500 simulated data sets closest to the observed data set and calculating the proportion that belong to each scenario (direct estimate) and (ii) performing a logistic regression on the closest 1% of data sets to the observed data (Cornuet et al. 2008). We evaluated the posterior distribution of estimates by performing a regression on the closest 1% of logit-transformed data sets and evaluated bias and precision of each parameter by calculating mean relative bias and relative mean square error using the standard procedures in DIY-ABC.

**Table 1:** Demographic scenarios, priors and posterior estimates used in approximate Bayesian computation (ABC) analyses of the Cousin Seychelles warbler population. Time is in generations (generation time = 4 years), CI = credible intervals, Bias = mean relative bias and RMSE = relative mean square error (Cornuet et al. 2008).

Parameter	Prior	Posterior estimates		Confidence in parameter estimation	
		Median	95% CI	Bias	RMSE
Scenario 1 (bottleneck)					
*N*_e_ (contemporary)	1–100	46	29–75	0.095	0.434
*N*_e_ (ancestral)	1–100,000	6,900	2,400–9,700	−0.032	0.585
Time (bottleneck)	5–500	55	33–64	0.176	0.498

For comparison, we also estimated *N*_e_ for the museum (Cousin) and contemporary populations using the Bayesian approach implemented in ONeSAMP (Tallmon et al. 2004), with prior minimum values set to 1 and maximum values set to 10 000 for the museum, and 320 for the contemporary population (current census size), respectively. To check how sensitive these estimates were to priors, we reran ONeSAMP for the contemporary population using the same, wide priors as for the museum population (1–10 000).

## Results

Of the 26 museum samples, 23 were successfully genotyped at 10 or more of the 12 loci. One sample from Marianne was genotyped at only three loci (museum ID 1876-574; Table S1) so was excluded from further analysis. Two were genotyped at six loci (USNM 119753 and AMNH 265502; Table S1); population-level analyses carried out both with and without these two samples yielded the same results (data not shown), so they were retained, leaving sample sizes of 19 and 6 for Cousin and Marianne, respectively, and a total sample size of 126 individuals when the 1997 and 2011 populations were included. The genotyping error rate in the museum specimens was 2.5%. Observed and expected heterozygosity for each locus and population, along with results of tests for deviations from HWE and null allele frequency estimates, are given in Table S4.

**Figure 2:**
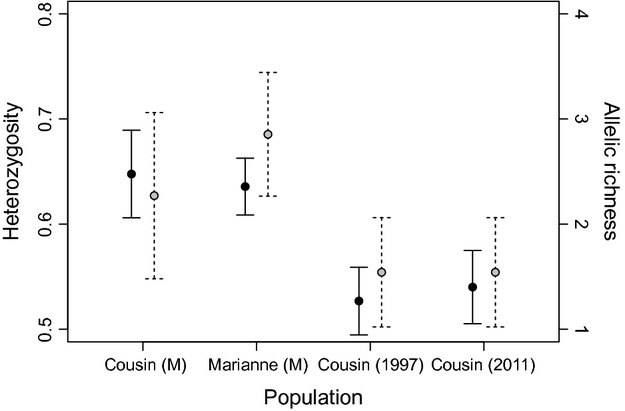
Expected heterozygosity (black dots and solid bars) and allelic richness (grey dots and dashed bars) averaged over 12 microsatellite loci in museum (M) and contemporary Seychelles warbler populations. Error bars represent standard error.

Pairwise *F*_ST_ estimates between museum and contemporary samples were moderately large and highly significant (all *F*_ST_* *> 0.1, *P *<* *0.001; Table2), but there was no significant differentiation between the 1997 and 2011 populations (*F*_ST_ = 0.001, *P *=* *0.2; Table2). A PCA suggested that the Cousin and Marianne museum samples formed two separate clusters, with the contemporary samples forming a separate third cluster (Fig.2). STRUCTURE analyses indicated three and two genetic clusters using the log likelihood and Δ*K* methods, respectively, which corresponded closely to the results from the PCA (Figure S1). The only difference between the PCA and STRUCTURE analyses was that STRUCTURE, when *K *=* *2, grouped the Marianne samples with the Cousin museum samples (Figure S1) – however, this is most likely the result of the low sample size on Marianne (Kalinowski 2011).

**Table 2:** Pairwise *F*_ST_ (below diagonal) and *D*_EST_ (above diagonal) in museum and contemporary Seychelles warbler populations.

	Marianne (M)	Cousin (M)	Cousin (97)	Cousin (11)
Marianne (M)	–	0.09	0.22	0.18
Cousin (M)	0.13	–	0.07	0.05
Cousin (1997)	0.28	0.12	–	**<0.001**
Cousin (2011)	0.27	0.11	**0.002**	–

Nonsignificant values (*P *>* *0.05) are highlighted in bold.

**Figure 3:**
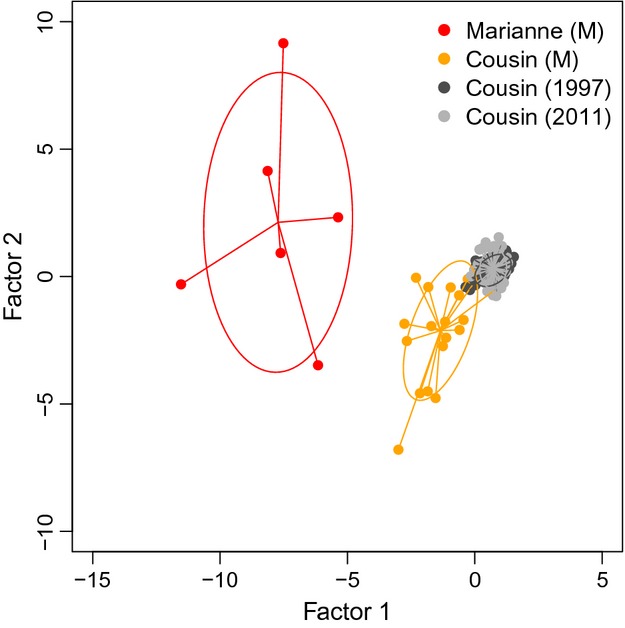
Principal components analysis of 126 Seychelles warbler samples, based on 12 microsatellite loci. Each point represents an individual, and ellipses represent 95% confidence limits for population-level groups.

In going from the museum to the contemporary populations, there was a 25% loss of allelic richness and a 19% loss of heterozygosity (Fig.3). There was significant heterozygosity excess in the Cousin museum population (*P *=* *0.001–0.004, depending on the proportion of stepwise mutations assumed) and in both the 1997 and 2011 populations (*P *<* *0.001 for both years, regardless of the mutation model). There was no evidence for heterozygosity excess in the Marianne museum populations (*P *=* *0.09–0.27), but the number of individuals (*n* = 6) is lower than the recommended minimum sample size for this analysis (*n* = 10), so this result must therefore be treated with caution. Mean (±SE) Garza and Williamson's *M* ratio was 0.72 ± 0.06 for the Marianne museum population, 0.81 ± 0.05 for the Cousin museum population and 0.66 ± 0.05 for both the 1997 and 2011 Cousin populations (*M *≤* *0.68 is indicative of a bottleneck; Garza and Williamson 2001). As *M* is expected to increase with sample size (Garza and Williamson 2001), these results are conservative. Together, these tests indicate that a bottleneck may have already occurred when the museum samples were obtained from Cousin, with strong evidence for a bottleneck in the contemporary samples.

Simulations carried out in DIY-ABC strongly supported a bottleneck over the scenario of a constant *N*_e_ over time (posterior probabilities = 1 and 0, respectively, for both direct and logistic regression estimates). We evaluated confidence in this scenario choice by simulating 500 pseudo-observed data sets under the constant *N*_e_ scenario, performing ABC analyses and calculating the number of times the bottleneck scenario was incorrectly chosen, and found that in no case did this occur (suggesting a Type 1 error rate of <0.002). That 14 of the 15 observed summary statistics were well within the range of the simulated data for the bottleneck scenario (Table S5) gives us further confidence in our scenario choice. Posterior estimates suggested that the Cousin population was reduced from an *N*_e_ of several thousand to <50 between 120 and 250 years ago, with relatively low bias and high precision estimates (Table1; assuming a generation time of 4 years). The estimate of ancestral *N*_e_ did have wide-ranging credible intervals – a result that could arise from low sample size in the museum population or a bottleneck occurring before the museum samples were taken. Estimates of *N*_e_ in ONeSAMP, like DIY-ABC, gave a much higher value for the museum population than for the contemporary population, although absolute estimates were consistently lower (museum population: mean = 268, 95% credible intervals = 175–1320; contemporary population: mean = 32; 95% credible intervals = 27–41). We note that sample sizes lower than 20 individuals can cause problems with ONeSAMP (D. Tallmon, personal communication) and therefore interpret absolute values from this program with caution.

## Discussion

The Seychelles warbler, like many bottlenecked species, is characterized by low levels of genetic diversity at neutral and functional genes (Richardson et al. 2000; Richardson and Westerdahl 2003; Hansson and Richardson 2005; Dawson et al. 2010); however, until now we have not had confirmation of exactly the timing, extent or duration of this bottleneck. Using museum samples, we found that prior to the 19th century, there was considerably more genetic diversity within and across the only two known ancestral Seychelles warbler populations. Moreover, we show that a substantial proportion of this genetic diversity was lost over subsequent decades. Simulations informed with the museum and contemporary population data indicate that the effective population size of the Seychelles warbler has historically been orders of magnitude greater than it is now.

The two main sources of genotyping error that can affect DNA-based studies of museum specimens are allelic dropout and false alleles (Taberlet et al. 1996; Wandeler et al. 2007). Our modest genotyping error rate of 2.5% in the museum specimens suggests that while genotyping error has most likely occurred in our museum specimens, it cannot be the explanation for the striking differences in genetic diversity and allelic composition between the historical and contemporary populations. Moreover, all available evidence suggests that the allelic dropout rate overwhelms the rate of false alleles in degraded samples (Wandeler et al. 2007; Arandjelovic et al. 2009), which in our case would cause an underestimation of the true prebottleneck diversity. With this evidence in mind, combined with the fact that we only included new alleles that were confirmed in replicated PCRs, we believe that our results can be interpreted as providing a minimum estimate of the loss of genetic diversity that has occurred in the Seychelles warbler as a result of the bottleneck.

There are very good records of the population size and status of the Cousin Seychelles warbler population from the 1960s onwards, but very little is known about the range and numbers of the species before then. The Seychelles are thought to have comprised a single, large island when sea levels were lower during the last ice age (Colonna et al. 1996; Rocha et al. 2013), and we therefore presume that the Seychelles warbler was widely distributed at that time. Our findings suggest that the warbler was indeed widely distributed until relatively recently, with the genetic consequences of the population decline occurring within the last century. The island of Cousin can support a maximum of approximately 350 birds (including juveniles); so the large ancestral effective population size estimated here must indicate that warblers were present on neighbouring islands, with gene flow occurring between populations. This at first seems counter-intuitive, as interisland dispersal is now rarely observed in the Seychelles warbler (Komdeur et al. 2004). However, in the past, when many more warblers were present across the Seychelles, rare dispersal events would easily have maintained genetic diversity across small island populations. The finding of moderate differentiation between the Marianne and Cousin museum populations (*F*_ST_ = 0.09) – two of the most geographically separated islands within the Praslin group (Fig.1) – is consistent with a scenario of limited, but not ubiquitous, historical dispersal between islands.

The loss of genetic diversity between museum and contemporary samples, the results from bottleneck tests, and the estimate of the bottleneck time from DIY-ABC all indicate a recent dramatic decline in population size in the Seychelles warbler. Most islands in the Seychelles were planted with coconuts in the late 19th and early 20th centuries, creating unsuitable habitat for Seychelles warblers (Komdeur 1994). This, along with the introduction of predatory rats and cats, would have been sufficient to drive steep declines across Seychelles warbler populations. Many other island species share a similar history to the Seychelles warbler, being extremely rare by the 20th century but with little or no information on prior population history – in the Seychelles, for example, there is the Seychelles magpie robin (*Copsychus sechellarum*), paradise-flycatcher (*Terpsiphone corvina*) and white-eye (*Zosterops modestus*; Collar and Stuart 1985). It is likely that many of these species have been subject to the same pressures and were also recently more widely distributed and abundant.

Long-term study systems such as the Seychelles warbler provide unique insight into key processes in ecology and evolutionary biology (Clutton-Brock and Sheldon 2010). Now having a historical backdrop against which questions can be addressed allows us to improve our interpretations of results. For example, the fact that female Seychelles warblers choose neither social nor extra-pair males to avoid inbreeding (Richardson et al. 2004) might be because inbreeding in this species has purged deleterious mutations, reducing selection for inbreeding avoidance. However, since it seems unlikely that inbreeding in the Seychelles warbler has been sufficiently severe or long-lasting enough to have purged all deleterious mutations, a more likely alternative explanation, given the large ancestral *N*_e_ documented here, is that inbreeding avoidance mechanisms might not evolve in larger populations where inbreeding is infrequent (Jamieson 2011). Likewise, although it has been suggested that in birds, bottlenecked island populations exhibit lower levels of extra-pair paternity (EPP) than outbred, mainland species (Griffith 2000), the Seychelles warbler does not fit this pattern, exhibiting higher levels of EPP (40% of offspring) than many outbred species (Richardson et al. 2001); however, if there is a genetic basis to promiscuity, and the warbler populations were large until recently, we do not necessarily expect low levels of EPP to have evolved.

The approaches used here, combining museum DNA with population simulations, have been and will be of use to other studies wishing to generate clear, testable hypotheses about ecological and evolutionary phenomena. These may be questions similar to those that we address using the Seychelles warbler system concerning inbreeding depression and the evolution of promiscuity. Alternatively, they may be questions related to older ecological and evolutionary phenomena, such as the role of climate fluctuations and prehistoric hunting pressures in historical population change (Fontaine and Snirc 2012) or the role of drift, mutation and selection in shaping patterns of genetic diversity (Yeung et al. 2011; Spurgin et al. 2014). And as more studies gain an understanding of population history, the generality of ecological and evolutionary hypotheses can be tested using comparative approaches. For example, the relationship between genetic diversity and EPP in birds is at present restricted to analysis of a few outbred species (Spurgin 2013). Comparing pre- and postbottleneck genetic diversity to EPP across a range of species would be a very promising approach to this question, as by doing so one could explore how responsive promiscuity is to sudden changes in population size.

Genetic diversity is crucial for the long-term persistence of populations and species and is one of the IUCN's three global conservation priorities along with species and ecosystem diversity (McNeely et al. 1990). The rapid loss of genetic diversity, such as that observed here, is therefore a cause for concern, as the evolutionary potential of this species has almost certainly been reduced. Species such as the Seychelles warbler, which have undergone the most rapid and severe reductions in population size, are likely to be at higher risk of extinction in the medium to long term. This is particularly concerning as inbreeding and genetic diversity have been shown to reduce fitness in the Seychelles (Brouwer et al. 2007, 2010). Some of the risks associated with low genetic diversity in this species will have been mitigated by the translocations. However, given that *N*_e_ was already dramatically reduced when the translocations were carried out, and the further bottlenecks associated with moving a subset of individuals (Wright et al. 2014), it is likely that genetic diversity is still being lost across the Seychelles warbler populations. With this in mind, and given that we now know that there was historical migration between warbler populations, there is a strong argument for carrying out assisted migration to preserve genetic diversity of this species. As more studies use museum specimens in a conservation context, we will be able to make better-informed conservation decisions. Most importantly, by identifying past changes in demography, it will be possible to identify those populations and species in greatest need of conservation action and to make evidence-based decisions about what action is most appropriate and how monitoring should be undertaken.

## Data archiving statement

Raw microsatellite data, along with data and scripts for R, have been deposited in the Dryad Digital Repository: http://doi.org/10.5061/dryad.km83c.

## Supporting Information

**Figure S1.** Results from STRUCTURE analyses on 126 Seychelles warbler individuals from museum (M) and contemporary populations. **Table S1.** Collection details of 26 museum specimens of Seychelles warbler along with the number of microsatellite loci each sample was genotyped at. **Table S2.** Primer and multiplex details for all loci tested in museum samples. **Table S3.** Nucleotide sequences for each previously unpublished primer tested in the museum samples. **Table S4.** Levels of genetic diversity, results of tests for Hardy–Weinberg equilibrium (HWE) and estimates of null allele frequencies at 12 microsatellite loci in museum (M) and contemporary Seychelles warbler populations. **Table S5.** Model checking for DIY-ABC analyses.
