# Supplementary material for: Museum DNA reveals the demographic history of the endangered Seychelles warbler
Source: Evol Appl. 2014 Aug 11;7(9):1134–43. doi: 10.1111/eva.12191 (PMC4231601; doi:10.1111/eva.12191)
Supplement: Supplementary file 1 — Figure S1. Results from STRUCTURE analyses on 126 Seychelles warbler individuals from museum (M) and contemporary populations. Table S1. Collection details of 26 museum specimens of Seychelles warbler along with the number of microsatellite loci each sample was genotyped at. Table S2. Primer and multiplex details for all loci tested in museum samples. Table S3. Nucleotide sequences for each previously unpublished primer tested in the museum samples. Table S4. Levels of genetic diversity, results of tests for Hardy–Weinberg equilibrium (HWE) and estimates of null allele frequencies at 12 microsatellite loci in museum (M) and contemporary Seychelles warbler populations. Table S5. Model checking for DIY-ABC analyses. [file eva0007-1134-sd1.docx]

**Supporting information**

**Appendix S1:** Phylogenetic analyses of malaria lineages detected in corncrake

We built a tree describing the phylogenetic relationships between the detected malaria lineages. We first assessed the model of sequence evolution with jModelTest 2.1.3 (Darriba et al., 2012). Following the AICc scores, we chose the GTR + gamma model of nucleotide substitution for further analyses. The phylogenetic tree was constructed using the Bayesian method implemented in MrBayes 3.2.1 (Ronquist and Huelsenbeck, 2003). We ran the analysis with 4 simultaneous Markov chains during 5 million generations sampled every 100 generations, and discarding 25% of the trees as burn-in. As an outgroup we included a cytochrome b sequence of *Plasmodium falciparum,* identified in gorillas (*Gorilla gorilla*) (Genbank accession number: GU045311.1). We used FigTree 1.4 (http://tree.bio.ed.ac.uk/software/figtree) to plot the resulting tree and reported the Bayesian support of tree nodes as computed by MrBayes (Figure S2).

**Appendix S2:** Estimation of effective population size by Approximate Bayesian Computation

We used an Approximate Bayesian Computation (ABC) (Beaumont *et al.* 2002) method previously implemented to investigate the demographic history of corncrake across Europe (Fourcade et al. *submitted*). For each sampling location, we simulated data using “ABCtoolbox” (Wegmann *et al.* 2010) to sample parameters in our prior distributions and “Fastsimcoal” (Excoffier & Foll 2011) for data simulation, under three simple demographic scenarios: decreasing, constant and increasing effective population size. One million simulations were conducted per demographic model and per population, and the most probable scenario was selected following the neural network approach implemented in the “abc” R package (Csilléry *et al.* 2012) using a 25% tolerance rate. Among the nine sampled populations, five were assigned to the model of decreasing effective population size and four to the constant model with a high posterior probability (0.80 ± 0.16 SD) (Fourcade et al. *submitted*).

Using the models selected in this previous work (Table S2), posterior probabilities of parameters were assessed following the neural network method. Effective/census population size ratio is usually around 0.10 in wild populations (Frankham 1995). Since the European corncrake population is estimated at ca. 3,000,000 birds (Schäffer & Koffijberg 2004), the prior distribution for effective population size was set between 1 and 1,000,000 individuals to cover all plausible values. The prior was log-transformed to allow better estimation of small sizes, and set uniformly between 1 and 6. Tolerance rates were set to 25%, *i.e.* the 250,000 simulations that are the closest to observed data were used to estimate parameters. In all analyses, neural network algorithms used 10 networks and five hidden layers. Parameters were logit-transformed to prevent extrapolation outside prior distributions.

Table S1: Grouping of loci into multiplexes, fluorescent labelling, and genetic diversity statistics calculated for each microsatellite locus. *N_A_*: number of alleles per locus, *H_o_*: observed heterozygosity, *H_e_*: expected heterozygosity or gene diversity, *F_IS_*: Wright’s inbreeding coefficient, *G_ST_* and *D*: two estimators of population differentiation. From Fourcade *et al.* (submitted).

| Locus | Multiplex | Dye | *N_A_* | *H_o_* | *H_e_* | *F_IS_* | *G_ST_* | *Jost's D* |
| --- | --- | --- | --- | --- | --- | --- | --- | --- |
| Crex1 | 3 | NED | 24 | 0.693 | 0.857 | 0.191 | 0.007 | 0.069 |
| Crex2 | 3 | FAM | 24 | 0.823 | 0.898 | 0.083 | 0.009 | 0.097 |
| Crex6 | 1 | HEX | 34 | 0.874 | 0.909 | 0.039 | 0.022 | 0.240 |
| Crex7 | 1 | FAM | 21 | 0.834 | 0.874 | 0.046 | 0.024 | 0.184 |
| Crex8 | 1 | NED | 24 | 0.832 | 0.902 | 0.078 | 0.003 | 0.041 |
| Crex9 | 1 | NED | 21 | 0.871 | 0.906 | 0.038 | 0.006 | 0.070 |
| Crex11 | 2 | NED | 33 | 0.890 | 0.919 | 0.031 | 0.019 | 0.236 |
| Crex12 | 2 | FAM | 24 | 0.603 | 0.852 | 0.292 | 0.006 | 0.072 |
| CAM18 | 2 | HEX | 16 | 0.616 | 0.601 | -0.025 | 0.013 | 0.021 |
| TG02-120 | 3 | FAM | 11 | 0.325 | 0.432 | 0.249 | 0.040 | 0.038 |
| TG04-012 | 3 | HEX | 9 | 0.653 | 0.659 | 0.008 | 0.008 | 0.017 |
| TG04-012a | 2 | FAM | 11 | 0.241 | 0.260 | 0.071 | 0.035 | 0.014 |
| TG04-041 | 1 | HEX | 12 | 0.725 | 0.748 | 0.031 | 0.002 | 0.009 |
| TG05-30 | 3 | HEX | 9 | 0.583 | 0.544 | -0.072 | 0.009 | 0.009 |
| TG012-015 | 1 | HEX | 13 | 0.580 | 0.668 | 0.132 | 0.003 | 0.013 |

**Table S2:** Posterior probability of demographic models in each sampling site, and for all data pooled together, inferred by the ABC analysis, according to Fourcade et al. (*submitted*). The selected model is shown in bold and has been used in *N_e_* calculation.

|  | Posterior probability | | |
| --- | --- | --- | --- |
|  | decreasing | constant | increasing |
| All data | **0.98** | 0.02 | 0.00 |
| France | **0.85** | 0.15 | 0.00 |
| Germany | **0.98** | 0.02 | 0.00 |
| Czech Republic | 0.12 | **0.88** | 0.00 |
| Poland (north) | **0.73** | 0.27 | 0.00 |
| Poland (south) | 0.46 | **0.54** | 0.00 |
| Poland (east) | **1.00** | 0.00 | 0.00 |
| Latvia | **0.91** | 0.09 | 0.00 |
| Belarus | 0.26 | **0.74** | 0.00 |
| Russia | 0.40 | **0.60** | 0.00 |

**Table S3:** Estimates of effective population size (mode of posterior distribution and 95% confidence intervals) inferred from Approximate Bayesian Computing, and mean local census size inferred by field surveys, from Schäffer & Koffijberg (2004) (minimum – maximum estimations), for all populations pooled and for each sampling population separately. In order to provide a fine estimation of small sizes, effective population sizes were estimated on a logarithmic scale (see Figure S2 for full posterior probabilities), the values shown are thus back transformed.

|  | | | Effective population size | | | Census size | | |
| --- | --- | --- | --- | --- | --- | --- | --- | --- |
| All Populations | 385833 | | (85225-744614) | 1650000 | | (1300000-2000000) ^1^ |  |  |
| France | 94812 | | (42983-244066) | 1150 | | (1102-1198) |  |  |
| Germany | 277179 | | (123777-732928) | 5100 | | (4000-6200) |  |  |
| Czech Republic | 155779 | | (91755-739527) | 3200 | | (3000-3400) |  |  |
| Poland (north) | 66101 | | (20111-446583) | 75000 | | (60000-90000) |  |  |
| Poland (south) | 137631 | | (83894-723401) | 75000 | | (60000-90000) |  |  |
| Poland (east) | 50976 | | (25787-364012) | 75000 | | (60000-90000) |  |  |
| Latvia | 111538 | | (61370-475166) | 64000 | | (52000-76000) |  |  |
| Belarus | 131327 | | (79565-742067) | 85000 | | (50000-120000) |  |  |
| Russia | 150069 | | (93545-735899) | 2500000 | | (2000000-3000000) ^2^ |  |  |

^1^ Estimation for the whole European population, from Birdlife International (2012)

^2^ Estimation for European Russia

**Figure S1:** Synthetic climatic predictor, obtained from the first axis of a PCA performed on a set of eight bioclimatic variables from the *Worldclim* project (Hijmans *et al.* 2005)**.** The contribution of the PCA axis to the total climatic variation is 50.16%.

**Figure S2:** Phylogenetic tree of the ten malaria lineages detected in eight corncrake populations (no malaria infection was detected in Germany) over Europe. *Plasmodium falciparum* is included as an outgroup. The Bayesian support of nodes is placed next to the nodes.

***References used in supporting information***

Beaumont MA, Zhang W, Balding DJ (2002) Approximate Bayesian computation in population genetics. *Genetics*, **162**, 2025–2035.

Birdlife International (2012) Crex crex. In: *IUCN Red List of Threatened Species Version 2012.1*. <www.iucnredlist.org>.

Csilléry K, François O, Blum MGB (2012) abc: an R package for approximate Bayesian computation (ABC). *Methods in Ecology and Evolution*, **3**, 475–479.

Excoffier L, Foll M (2011) Fastsimcoal: a Continuous-Time Coalescent Simulator of Genomic Diversity Under Arbitrarily Complex Evolutionary Scenarios. *Bioinformatics*, **27**, 1332–4.

Fourcade Y, Richardson DS, Keišs O et al. Range dynamics at the continental scale: a test of the central-marginal hypothesis in the Corncrake, a species with a complex conservation status. *Submitted manuscript*

Frankham R (1995) Effective population size/adult population size ratios in wildlife: a review. *Genetical Research*, **66**, 95–107.

Hijmans RJ, Cameron SE, Parra JL, Jones PG, Jarvis A (2005) Very high resolution interpolated climate surfaces for global land areas. *International Journal of Climatology*, **25**, 1965–1978.

Schäffer N, Koffijberg K (2004) Crex crex Corncrake. *Bwp Update*, **6**, 57–78.

Wegmann D, Leuenberger C, Neuenschwander S, Excoffier L (2010) ABCtoolbox: a versatile toolkit for approximate Bayesian computations. *BMC Bioinformatics*, **11**, 116.
